# The SNARE protein family of *Leishmania major*

**DOI:** 10.1186/1471-2164-7-250

**Published:** 2006-10-06

**Authors:** Sébastien Besteiro, Graham H Coombs, Jeremy C Mottram

**Affiliations:** 1 Wellcome Centre for Molecular Parasitology and Division of Infection & Immunity, Institute of Biomedical and Life Sciences, University of Glasgow, G12 8TA, UK

## Abstract

**Background:**

*Leishmania major *is a protozoan parasite with a highly polarised cell shape that depends upon endocytosis and exocytosis from a single area of the plasma membrane, the flagellar pocket. SNAREs (soluble *N*-ethylmaleimide-sensitive factor adaptor proteins receptors) are key components of the intracellular vesicle-mediated transports that take place in all eukaryotic cells. They are membrane-bound proteins that facilitate the docking and fusion of vesicles with organelles. The recent availability of the genome sequence of *L. major *has allowed us to assess the complement of SNAREs in the parasite and to investigate their location in comparison with metazoans.

**Results:**

Bioinformatic searches of the *L. major *genome revealed a total of 27 SNARE domain-containing proteins that could be classified in structural groups by phylogenetic analysis. 25 of these possessed the expected features of functional SNAREs, whereas the other two could represent kinetoplastid-specific proteins that might act as regulators of the SNARE complexes. Other differences of *Leishmania *SNAREs were the absence of double SNARE domain-containing and of the brevin classes of these proteins. Members of the Qa group of *Leishmania *SNAREs showed differential expressions profiles in the two main parasite forms whereas their GFP-tagging and *in vivo *expression revealed localisations in the Golgi, late endosome/lysosome and near the flagellar pocket.

**Conclusion:**

The early-branching eukaryote *L. major *apparently possess a SNARE repertoire that equals in number the one of metazoans such as *Drosophila*, showing that the machinery for vesicle fusion is well conserved throughout the eukaryotes. However, the analysis revealed the absence of certain types of SNAREs found in metazoans and yeast, while suggesting the presence of original SNAREs as well as others with unusual localisation. This study also presented the intracellular localisation of the *L. major *SNAREs from the Qa group and reveals that these proteins could be useful as organelle markers in this parasitic protozoon.

## Background

Eukaryotic cells contain many internal organelles surrounded by membrane boundaries, where specialised and essential functions are performed. The traffic between these different organelles is mainly mediated by vesicular transport [1]. The mechanism required for this type of transport involves a complex and specifically regulated machinery that allows budding of vesicles from a donor compartment, followed by their translocation to their target, to which they have to dock and then fuse. Among the lipid and protein factors that are thought to be involved in these processes, a large family of proteins called soluble *N*-ethylmaleimide-sensitive factor (NSF) adaptor proteins (SNAPs) receptors (SNAREs) are considered essential (see [2-4] for a review).

SNAREs were originally classified according to the membrane component where they were required, as v-SNAREs (associated with the vesicles) or t-SNAREs (associated with the target compartment) [5]. These proteins have a helical structure and the interaction between v-SNAREs and t-SNAREs leads to the formation of a *trans*-SNARE complex consisting of four SNARE motifs in a parallel four-helical bundle catalysing the docking and fusion of the vesicle with the target compartment [6,7]. Tethering factors and regulators such as Rab or Sec1/Munc family of proteins allow a fine spatial and temporal control of SNARE-mediated fusion and might as well monitor specificity [8]. One possible contradiction with the original SNARE hypothesis is that the same SNARE might be involved in several targeting events and be required either on a vesicle or a target membrane [9]. Thus, rather than being functionally classified in v- and t-SNAREs, these proteins can also be structurally distinguished. Indeed, the SNARE motif involved in the formation of the helical bundle of the SNARE complex is conserved but bears unique features allowing the classification as Q- and R-, according to the residue present in the centre of the motif [10]. The Q group can be further divided into three sub-groups according to their overall homology in the SNARE domain: Qa (or syntaxins), Qb (or SNAP N-terminal) and Qc (or SNAP C-terminal) [11].

Kinetoplastid parasites, such as *Trypanosoma *or *Leishmania *are very polarised cells that contain a dense and complex membrane network around the flagellar pocket, an invagination of the plasma membrane where the flagellum emerges from the cell body and where most of the exchanges with the external milieu occur [12]. The exocytic and endocytic pathways are contained within the anterior region of the *Leishmania *cell and the polarised organelles include the endosomal and lysosomal systems, the Golgi complex, but not the endoplasmic reticulum (ER) that is distributed throughout the cytoplasm. Kinetoplastids are among the earliest-branching eukaryotes possessing a mitochondrion. Indeed, recent analyses combining taxon-rich nuclear small subunit rRNA gene trees and protein phylogenies confirm that they are related to euglenoids [14], with whom they form one of the eight eukaryotic group called discicristates [15]. Thus, kinetoplastids constitute an interesting model to study the features of the vesicular and membrane transport systems that could have been conserved from more primitive eukaryotes [13]. More importantly, the very function of intracellular traffic has obvious implications for parasites, which depend extensively on endocytic and exocytic pathways, whether it is to get nutrients from the host or secrete virulence factors.

The recent availability of the complete genome sequence from *Leishmania major *[16] has allowed us to conduct a genome-wide analysis of the potential SNAREs present in the parasite. We have particularly focused on the members of the syntaxin subfamily to analyse their expression and, using GFP-tagged expression, elucidate their location within *Leishmania*.

## Results and discussion

### Classification of SNARE domain-containing *L. major *predicted proteins

**Figure 1:**
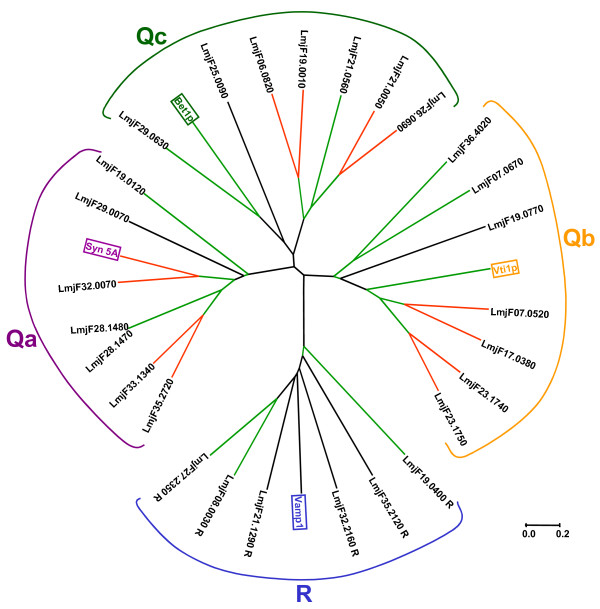
**Unrooted tree of *L. major *SNARE domain-containing proteins**. A 71 amino acids-long SNARE motif (see Figure S1 from Additional file 1) from *L. major *predicted SNARE proteins was aligned using the Clustal W algorithm and a phylogenetic tree was generated as described in the Methods section. Selected characterised members from the Qa, Qb, Qc and R groups were included in the analysis (boxed). They are, respectively, human Syntaxin 5A (NP_012262), yeast Vti1p (Q04338), yeast Bet1 (NP_012262) and human Vamp1 (CAA88760). Branches with bootstrap values > 45 in green and > 90 in red. The scale bar represents 0.2 mutational changes per residue (20 PAM units). *L. major *sequences are labelled with their identifier in the GeneDB database [39].

As SNARE proteins share a conserved functional domain, we used sequence homology searches to identify proteins bearing this SNARE coiled-coil domain in the *L. major *predicted proteome. We found 27 putative proteins and used a phylogenetic analysis to classify them after multiple sequence alignments of their SNARE domain [10,11]. When analysed, the *Leishmania *sequences segregated to four different groups (Qa, Qb, Qc and R, Fig. 1) in accordance with genome-wide classifications performed with other organisms [2,11,17]. The identification of each structural group was performed by including sequences from already characterised members of the Qa (human syntaxin 5a) Qb (yeast Vt1p), Qc (human Bet1) and R (human Vamp1) groups. Group clustering was then further checked by including more sequences from characterised human or yeast proteins and performing amino acid alignments of the SNARE domains (Additional file 1, Fig. S1A–D). We could then confirm that the Qa group, bearing members of the syntaxin family (see [18] for a review), is comprised of seven proteins in *Leishmania*, that the same number of proteins are clustered in each of the Qb and Qc groups, whereas the R group comprises six members.

### General and structural features of *L. major *SNAREs

The *L. major *putative SNAREs generally appear to have the conserved features observed for these proteins (see Additional file 2 and Figure S2 of Additional file 1). Most of them are small (between 100–360 amino acids), and bear a C-terminal hydrophobic region that could act as membrane-anchoring domain. A few proteins (LmjF28.1480, LmjF33.1340, LmjF32.2160 and LmjF35.2120) do not have any predicted transmembrane domain or GPI-anchor and thus might rely on a different process to bind to the membranes. Indeed, LmjF33.1340 and LmjF35.2120 have predicted prenylation sites (in positions 272 and 202, respectively) and the same C-terminal cysteine residue of LmjF35.2120 could also be palmitoylated. Protein lipidation has been shown before to be a way of linking SNAREs to membranes, for example the human SNAREs syntaxin 11 [19] and Ytk6, can be both prenylated and palmitoylated [20]. The other two proteins lacking predicted transmembrane domains (LmjF28.1480, LmjF32.2160), also appear to be lacking putative lipidation sites, so their membrane attachment may be mediated by binding to another SNARE, as suggested for human SNARE SNAP-25 [21].

All the identified sequences contained a single SNARE motif, generally located at the C-terminal end of the proteins. Animals, higher plants and fungi contain SNAP-25-like proteins bearing two SNARE motifs, at their N- and C-termini (i.e. SNAP-25, SNAP-23 and SNAP-29 in humans, Sec9p and Spo20p in the yeast *Saccharomyces cerevisiae*). Their N-terminal motif belongs to the Qb group and the C-terminal one to the Qc group. However, we could not identify any protein containing two SNARE motifs in *L. major*, which is consistent with the observation made previously for other early-branching eukaryotes such as *Trypanosoma brucei *and the diplomonad *Giardia lamblia *[13].

Most of the putative SNAREs we identified were found to possess specific N-terminal domains (see Additional file 2 and Figure S2 of Additional file 1). The Qa/syntaxins group is usually characterised by the presence of a N-terminal helical bundle (named Habc, as it contains three helical regions Ha, Hb and Hc) that can in some cases fold back into a closed conformation to interact with the SNARE motif [22,23] and be opened by regulator proteins. Using secondary structure prediction programs, we could identify the presence of such a bundle in all the members of the Qa group of *L. major *(see Additional file 2 and Figure S2 of Additional file 1). A similar series of helixes could as well be unambiguously predicted in sequences from *L. major *SNARE members of the Qb and Qc groups (see Additional file 2 and Figure S2 of Additional file 1). This is not surprising, as human syntaxin 6 (belonging to the Qc group) and vti1 (belonging to the Qb group) have also been shown to possess this feature [22,24]. Another characteristic feature identified in *L. major *SNAREs was found in the members of the R group. R-SNAREs can be subdivided into short VAMPs (vesicle-associated membrane proteins), also called brevins, and long VAMPs, or longins, depending on whether they contain a short and variable domain or a conserved longin domain (of up to 150 amino acids) at their N-terminus [25]. Brevins seem to be absent from the *L. major *SNARE repertoire, as indeed they are from *Plasmodium falciparum *and *Arabidopsis thaliana *[25]. All of the six identified *L. major *proteins with a putative R-SNARE domain possessed a N-terminal extension, which could be clearly identified, for most of them, through sequence homology with the longin domain (see Additional file 2 and Figure S2 of Additional file 1).

### SNARE-interacting proteins

The N-terminal domains of SNAREs are known to be involved in their regulation through interaction with several factors. As SNAREs play a crucial role in membrane fusion, these events need to be tightly regulated in space and time and a variety of SNARE-interacting proteins have already been identified. We searched the *L. major *genomic database and identified some of the key factors that might help regulate the SNARE machinery (Table 1).

Some of the most essential regulators of SNARE complexes are the human SNAP and NSF proteins (Sec17 and Sec18 in *S. cerevisiae*). NSF is an AAA-ATPase that functions as an hexameric complex to dissociate the SNARE complex through hydrolysis of ATP [26,27]. NSF is bound to the SNARE complex through an association with several α-SNAP proteins whose positively charged residues are linked to acidic charges of the SNARE complex [28]. Humans also express a neurons-specific β-SNAP isoform and a related ubiquitous γ-SNAP, which might not be able to interact directly with the SNARE complex but interacts with NSF [29]. The *L. major *database contains a putative NSF (LmjF20.0810) bearing a predicted AAA-ATPase domain (residues 254–453). We also found two isoforms of the α/β-SNAP family (LmjF20.1690, LmjF32.2890) and one orthologue of γ-SNAP (LmjF34.0540). This would be consistent with the presence of well-conserved SNARE complex dissociation machinery in *L. major*.

Another family of SNARE-regulating proteins is the Sec1/Munc18 (SM) family, for which there is still controversy as to their putative SNARE-inhibiting or activating function [30]. However, recent data shows a potential role for mouse Munc18-1 in establishing a strongly tethered state of the SNARE complex and also in regulating the vesicle delivery rate [31]. The mode of interaction of SM proteins with their cognate SNARE proteins also appears to be complex, and there could be distinct mechanisms at different stages in the SNARE assembly/disassembly cycle [32]. Proteins of the SM family appear to function at different intracellular membrane compartments, have generally a high specificity for one SNARE protein and are thought to act as chaperone-like molecules [30]. For example, *S. cerevisiae *has four SM proteins: Sec1p, located at the plasma membrane; Sly1p, involved in *cis*-Golgi traffic; Vps45p, involved in *trans*-Golgi traffic; Vps33p, located at the vacuole/lysosome. In *L. major*, we found five putative SM proteins: one Sly1-like, two Vps45-like, one Vps33-like and one Sec1/Munc18-like. This suggests that all the classes of SM proteins are present in *L. major*.

There are other proteins that have been shown to interact with SNAREs and several of which are present only in certain species, or even specialised cell types in metazoans. Most of these factors could not be identified in *L. major*, but some proteins orthologous to human SNARE-interacting proteins, including a putative epsin [33] or a GATE-16 orthologue [34] could be found (LmjF25.0670 and LmjF25.0670, respectively). Similarly, orthologues to SNARE-interacting proteins from *S. cerevisiae *known as DNA-damage inducible protein DDI1/Vsm1 [35] and members of the Vtc complex [36], are apparently also present in the *L. major *genome.

### The Qa/syntaxin sub-family: gene expression

Characterisation of a neuronal endosomal SNARE complex showed it contained Syn7 (Qa), Vti1b (Qb), Syn8 (Qc) and VAMP8 (R) [37] and it is now generally believed that one member of each SNARE motif group (Qa, Qb, Qc, R) is involved in the formation of a single SNARE complex. Thus, we analysed more thoroughly members of the Qa/syntaxin sub-family only, to gain some general insights on the expression or localisation of *Leishmania *SNAREs.

**Figure 2:**
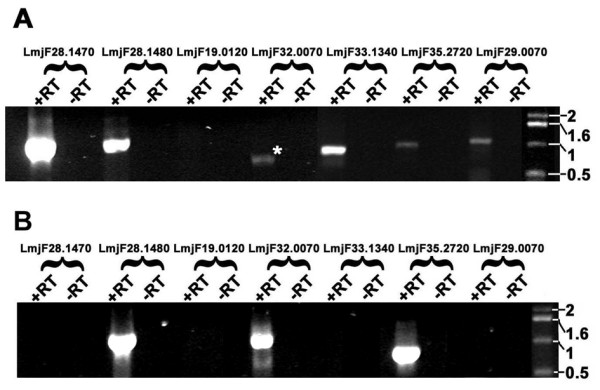
**Expression profile of mRNAs from *L. major *syntaxins**. Fragments obtained after semi-nested RT-PCR reactions on cDNA obtained from total RNA extracted from *L. major *promastigotes (A) or amastigotes (B). The fragment sizes are indicated on the right (in kb); -RT denotes that the reverse-transcriptase was not included in the initial reaction. The 5' primer used was specific for the "splice-leader" sequence, whereas the 3' primers were specific of each individual members of the Qa group. The star denotes an artefactual product.

First, to determine if the genes coding for the members of the Qa group were transcribed, we isolated total RNA from *L. major *promastigote and amastigote forms and performed semi-nested reverse transcriptase-PCR (RT-PCR) using a mRNA-specific "splice-leader" (SL) primer to amplify syntaxin transcripts (Fig. 2). In kinetoplastids, one of the major mechanisms regulating gene expression is at the level of translation [38], thus the presence of mRNA does not systematically account for the expression of the corresponding protein. However, we could detect transcripts for all genes belonging to the Qa group in *L. major *promastigotes (Fig. 2A) and/or amastigotes (Fig. 2B), except *LmjF19.0120*, which might be expressed at a very low level, or in another developmental form. The identity of the DNA fragments was confirmed by sequencing or restriction digest and all were as expected except for *LmjF32.0070 *in promastigotes, which was a PCR artefact (* in Fig 2A). *LmjF28.1470, LmjF29.0070 *and *LmjF33.1340 *expression was only detected in promastigotes, *LmjF32.0070 *only in amastigotes, *LmjF28.1480 *and *LmjF35.2720 *in both life cycle stages. Overall, these data suggest that, rather than being constant and ubiquitous, the expression of some of the SNAREs in *Leishmania *could be stage-regulated, which might reflect different trafficking requirements of different forms of the parasite.

The analysis of the mRNA-derived PCR products also allowed us to determine the SL addition site and therefore the likely ATG start codon for several of the genes. This was then compared with the automatic annotation carried out on the *L. major *genome [39]. A putative upstream in-frame start codon could be found for syntaxin 1-like gene *LmjF28.1470*, suggesting the production of a corresponding protein that would be longer than the one originally predicted. This is of importance as the additional N-terminal sequence lengthens the first two of the Habc helixes that are proposed to have a role in the targeting and function of syntaxin 1 [23,40]. In contrast, the product corresponding to *LmjF28.1480 *yielded a putative first start codon that was followed by three early stop codons shortly after, suggesting that the corresponding extended protein would not be produced and that *LmjF28.1480 *could be a pseudo gene. Finally, sequencing of the 5' end of the product obtained for *LmjF32.0070 *in amastigotes identified a start codon potentially used that is upstream of the one annotated in the *L. major *genome. However, the N-terminal extension of the predicted protein, in this case, did not significantly modify the structure of the Habc helixes as verified by software analysis (data not shown).

### The Qa/syntaxin sub-family: protein locations

To identify the intracellular locations of SNARE complexes in *L. major*, we systematically tagged with GFP the N-terminal end of the seven Qa members that we have identified and expressed them in *L. major *promastigotes in order to localise them by fluorescence microscopy. Such an approach has been used successfully before, for example to determine the localisation of *A. thaliana *SNAREs [17]. The seven genes coding for putative Qa members were amplified from *L. major *genomic DNA by PCR, cloned into a GFP-fusion expression vector [41], transfected into *L. major *promastigotes. Expression of the predicted fusion proteins for each gene was confirmed by Western blot with an anti-GFP antibody (Figure S3 from Additional file 1) and the cells were then observed by fluorescence microscopy. As overexpression of the fusion proteins can potentially lead to a mistargeting to improper locations, great care was taken in analysing the localisations and looking for consistency of the obtained signals in cells displaying either weak or strong fluorescence.

**Figure 3:**
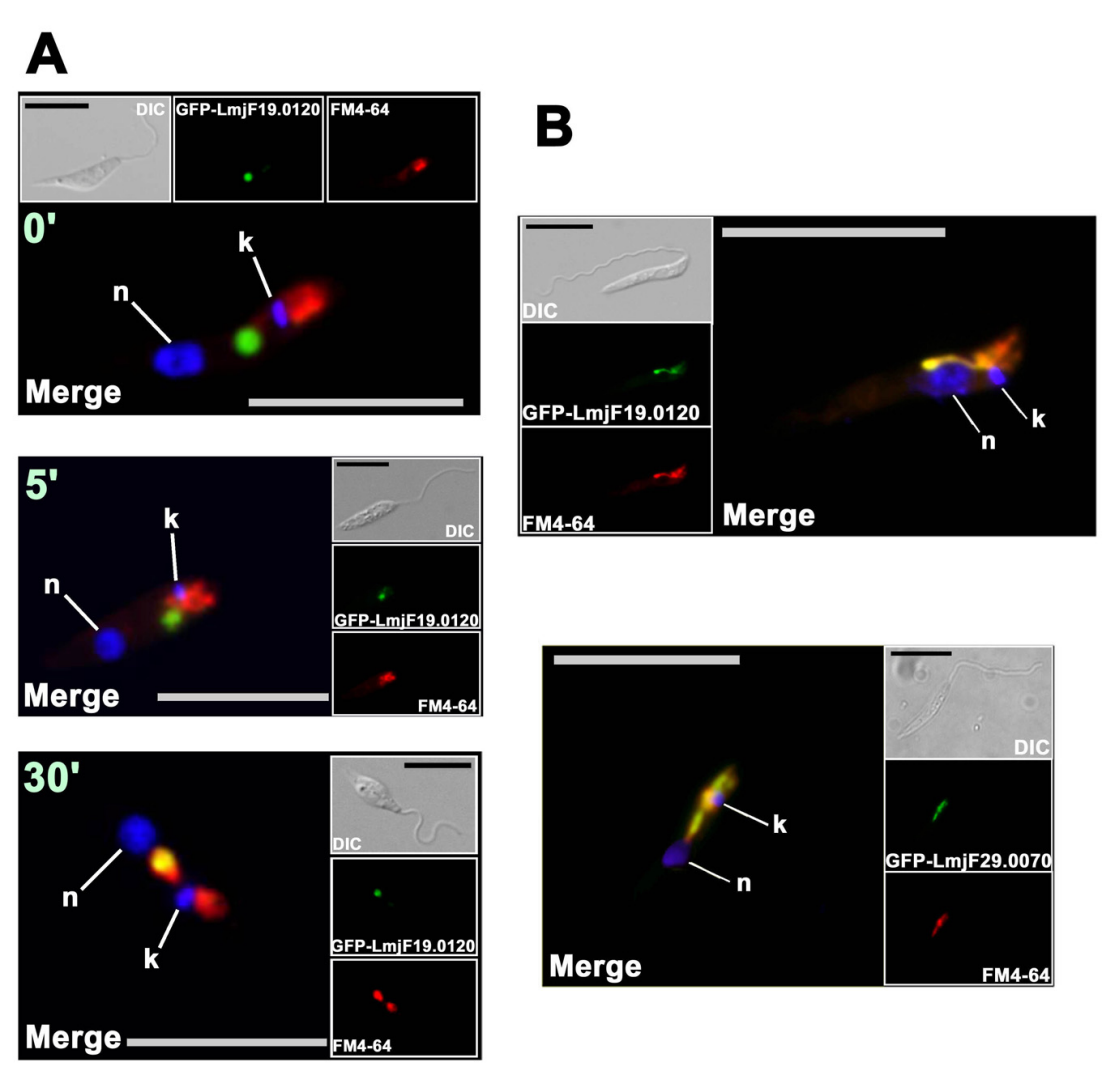
**GFP-LmjF19.0120 and GFP-LmjF29.0070 localise to the endo-lysosomal pathway in *L. major***. (A) Co-localisation of GFP-fused LmjF19.0120 with fluorescent endo-lysosomal tracer FM4-64 after incubation for increasing amounts of time (top) with live *L. major *promastigotes. (B) Localisation of GFP-LmjF19.0120 (top) and GFP-LmjF29.0070 (bottom) in a tubular compartment in live *L. major *stationary-phase promastigotes. Differential interference contrast (DIC) image, GFP fluorescence image (green) and the signal given by FM4-64 (red) after 30 minutes of incubation are presented in the insets and the two fluorescent signals are shown merged together with the DAPI staining of DNA (blue; n: nucleus; k: kinetoplast, mitochondrial DNA) in the enlarged images. Co-localised signal is shown in yellow. Scale bar = 10 μm.

A variety of fluorescence patterns were obtained and co-localisation with established cellular markers was used to tentatively identify the labelled compartments. With the exception of LmjF28.1480, all the constructs yielded localised signals, consistent with a presence in organelles of a vesicular nature and the probable membrane association of *L. major *Qa SNAREs. GFP-fused LmjF19.0120 and LmjF29.0070 produced similar types of signals, they were usually found to label a vesicular population concentrated at the anterior part of the cell (Fig. 3A). We used the lipophilic marker FM4-64 as an endocytic tracer in the GFP-LmjF19.0120-expressing cell line and found that after short incubation times (5–10 minutes), the GFP signal and FM4-64 were not co-localising, whereas longer incubation times (20 minutes-1 hour) with the dye led to a co-localisation (Fig. 3A). This suggests that the GFP-fused protein was present in the late endosomes or lysosomes. Similar results were obtained with the cell line expressing GFP-LmjF29.0070 (data not shown). Furthermore, in stationary-phase cultures from both cell lines, the signal could sometimes be seen extending in a characteristic tubular shape that was co-localising with FM4-64 (Fig. 3B). This probably represented a tubular type of endosome or the MVT-lysosome compartment [42,43].

**Figure 4:**
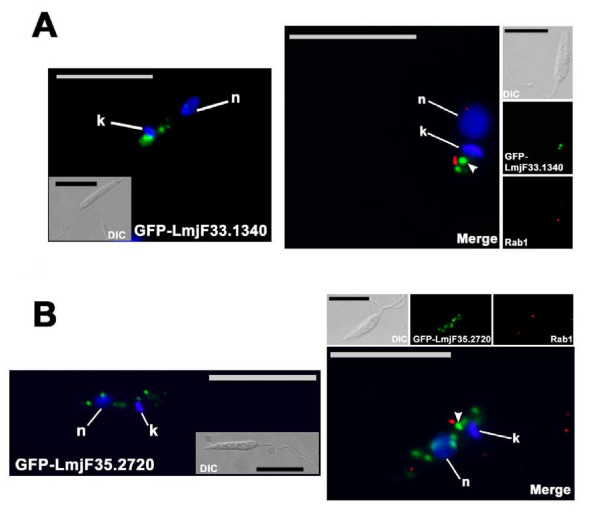
**GFP-LmjF35.2720 and GFP-LmjF33.1340 label punctate structures**. Localisation of GFP-fused LmjF33.1340 (A) and LmjF35.2720 (B) in live (left hand side) or fixed (right hand side) *L. major *promastigotes. Corresponding DIC images are shown; the GFP fluorescence signal is shown in green and immuno-labelling with Golgi marker Rab1 is shown in red. The merged fluorescent signals, together with the DAPI staining of DNA (blue; n: nucleus; k: kinetoplast) are shown in the enlarged image. Arrowheads point the predominantly fluorescent signal located close to the Golgi staining. Scale bar = 10 μm.

GFP-fused LmjF33.1340 and LmjF35.2720 displayed comparable signals, consisting of a variable number of puncta located throughout the cell body and often displaying a strong labelling at the anterior part of the cell, close to the mitochondrial DNA (also called the kinetoplast) (Fig. 4, arrowheads). LmjF33.1340 was generally found to display less of these puncta than LmjF35.2720 (Fig.4, compare A and B). As both proteins are somewhat similar in sequence to syntaxin 16 (see Additional file 2), which is a Golgi syntaxin, and as the Golgi apparatus is known to be located at the anterior part of the cell in kinetoplastids, we performed co-localisation studies with an antibody raised against *T. brucei *Rab1, a characterised kinetoplastid Golgi apparatus marker [44]. The results show that, although closely located, the syntaxins and Rab1 are in distinct locations (Fig. 4, right). Kinetoplastids possess a single Golgi apparatus, but as this organelle has a complex architecture with multiple cisternae and a typical *cis-trans *organisation, the GFP-fused proteins could still be in a part of the Golgi where Rab1 is not present. The complex signal pattern, however, could be suggestive of a localisation within multiple cellular compartments. The presence of some punctate labelling in a part of the cell between the kinetoplast and the flagellar pocket zone might suggest an association with early endosomal compartments, but the lack of co-localisation with endocytosed dye FM4-64 (data not shown) argue against this. The other GFP-stained structures in the rest of the cell body also remained unidentified, as no co-localisation could be shown with markers of the vesicular organelles acidocalcisomes (vacuolar proton pyrophosphatase, V-H^+^-PP [45]) and glycosomes (phosphofructokinase, PFK [46]) (data not shown).

**Figure 5:**
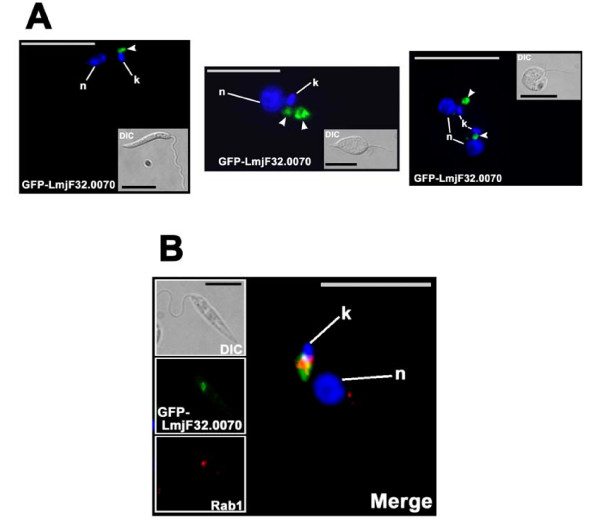
**GFP-LmjF32.0070 is associated with the Golgi**. (A) Localisation of GFP-fused LmjF32.0070 in live *L. major *promastigotes (insets display the corresponding DIC images). Arrowheads point the GFP-labelled structure(s) in a single promastigote (left), or in a dividing cell before (middle) or during (right) cytokinesis. (B) Co-localisation of GFP-LmjF32.0070 (green) and immuno-labelled Golgi protein Rab1 (red) in fixed *L. major *promastigotes. The merged signals, together with the DAPI staining of DNA (blue; n: nucleus; k: kinetoplast) are shown in the enlarged image. Co-localised signal is shown in yellow. Scale bar = 10 μm.

GFP-fused LmjF32.0070 was found in a compartment next to the kinetoplast (Fig. 5A). The labelled structures appeared to be duplicated in dividing cells before segregating during cytokinesis (Fig. 5A), similarly to what was observed for the generation of a new Golgi stack in dividing trypanosomes [47]. Moreover, when the cells were also stained for Rab1, there was a close association of the two signals (Fig. 5B), although they do not totally overlap. Hence, it appears that GFP-LmjF32.0070 is present in the Golgi system but might not be in the same sub-compartment as Rab1. This correlates well with the fact that LmjF32.0070 appears to be a *Leishmania *orthologue of syntaxin 5 (see Additional file 2), a Golgi syntaxin.

**Figure 6:**
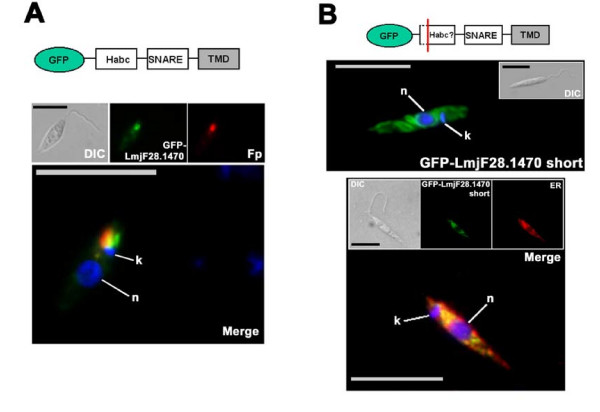
**GFP-fused LmjF28.1470 chimeras localise to different cellular compartments**. (A) Top; schematic of the structure from the GFP-fused LmjF28.1470. Bottom; localisation of GFP-fused LmjF28.1470 in live *L. major *promastigotes (DIC image shown on the top-left). The GFP fluorescence signal is shown in green and the flagellar pocket (Fp), labelled by FM4-64 at 4°C is shown in red. The merged signals, together with the DAPI staining of DNA (blue; n: nucleus; k: kinetoplast) are shown in the enlarged image. Co-localised signal is shown in yellow. Scale bar = 10 μm. (B) Top; schematic of the structure from the GFP-fused short version of LmjF28.1470. Middle; localisation of GFP-fused short LmjF28.1470 in live *L. major *promastigotes (inset shows DIC image). The GFP fluorescence signal is shown in green merged together with the DAPI staining of DNA in blue (n: nucleus; k: kinetoplast). Bottom; localisation of GFP-fused short LmjF28.1470 in fixed *L. major *promastigotes. Insets show the DIC image, the GFP fluorescence signal in green and immuno-labelling with ER marker LCB2 in red. The merged fluorescent signals, together with the DAPI staining of DNA (blue; n: nucleus; k: kinetoplast) are shown on the enlarged image. Co-localised signal is shown in yellow. Scale bar = 10 μm.

When the GFP-fused predicted syntaxin 1 homologue LmjF28.1470 (Fig. 6A, top panel) was expressed, all the cells displayed a very localised GFP signal at the very end of the anterior part of the cell body (Fig. 6A, bottom panel), and some cells also showed in addition an ER-like reticulated staining pattern. We used the lipophilic tracer FM4-64, known to accumulate in the flagellar pocket of kinetoplastids when incubated at 4°C [48], to label this compartment. Co-localisation between the GFP-LmjF28.1470 and FM4-64-labelled flagellar pocket revealed that the two signals were in distinct locations, but closely associated (Fig. 6B bottom panel). This would be indicative of the presence of the GFP-fused protein in a compartment spatially close to the flagellar pocket. Given the highly polarised organisation of the trafficking in kinetoplastids around the flagellar pocket, this would be consistent with a role of LmjF28.1470 in the cellular exchanges with the external milieu. Cells transfected with the construct producing the GFP-fused short version of LmjF28.1470 (Fig. 6B, top panel) displayed a reticulated signal of internal membranes, especially dense in the perinuclear zone (Fig. 6A, middle panel). In kinetoplastids, the ER comprises a nuclear envelope and a connected system of cisternal or tubular membranes that extends throughout the cell body. We performed co-localisation experiments between GFP-short LmjF28.1470 and ER-resident protein *Lm*LCB2 [49] and found that there was an extensive co-localisation of the two signals (Fig. 6A, bottom panel). This would be consistent with a localisation of this version of GFP-LmjF28.1470 in a sub-compartment of the ER. The N-terminal series of Habc helixes from syntaxin 1 is proposed to be able to bind the coiled-coil region of the SNARE motif to prevent the protein from interacting with unwanted partners during its trafficking to the plasma membrane [23,40]. In a mammalian cell type not expressing the SNAP-25 SNARE protein, N-terminally-truncated chimeras of syntaxin 1A localise to the ER, whereas the full-length protein is targeted to the plasma membrane [40]. This would appear to be similar in *L. major*, as the parasite lacks SNAP-25 homologues and we could localise the short form of LmjF28.1470 in the ER.

**Figure 7:**
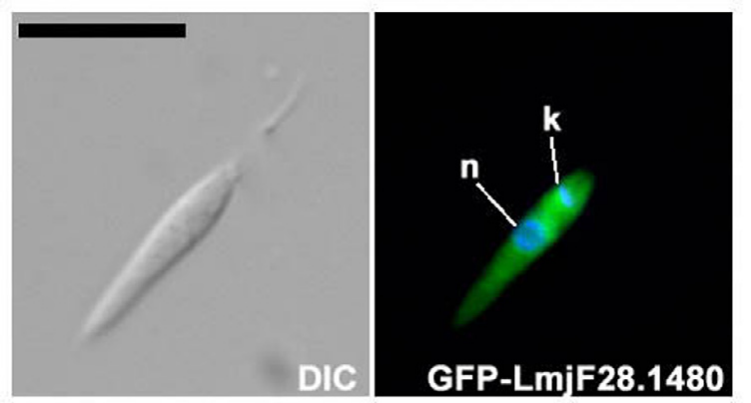
**GFP-LmjF28.1480 localises to the cytosol**. Localisation of GFP-fused LmjF28.1480 in live *L. major *promastigotes (DIC image shown on the left). The GFP fluorescence signal is shown in green merged together with the DAPI staining of DNA in blue (n: nucleus; k: kinetoplast). Scale bar = 10 μm.

Finally LmjF28.1480, which only differs from the short LmjF28.1470 in its C-terminal part with the absence of a predicted transmembrane domain, displayed a cytoplasmic fluorescent signal once fused with GFP (Fig. 7). No prenylation or palmitoylation site could be predicted for this protein (see Additional file 2) and the localisation of GFP fusions seems to confirm that it is not membrane-associated.

For several GFP-fusion proteins, some cells had labelling in more than one compartment. For instance, in some cells GFP-short LmjF28.1470 could apparently be seen in the Golgi in addition to the ER and, in a few cases, GFP-LmjF19.0120 was present on the plasma membrane in addition to the endo-lysosomal system. Clearly, these could be artefactual mislocalisations due to an overexpression of the GFP-fused proteins, but might also represent the fact that some SNAREs, although present in main organelle at steady-state levels, are cycling between two different compartments.

### Identifying *L. major *SNAREs

When putative homologues of the *L. major *SNAREs were searched for using BLAST, the returned results yielded E values generally higher than 10e^-20 ^(see Additional file 2). In most cases, the homology was significant within the SNARE domain, but not throughout the whole sequence. The analysis of the SNARE domain allowed us to classify *L. major *SNAREs into groups, but that is not sufficient to assign a function and a localisation to each individual SNARE. The localisation itself can be an additional clue to the identification of a SNARE, but as seen with the systematic GFP tagging of the members of the Qa group, sometimes it does not clearly match the expected BLAST result, such as with LmjF33.1340 and LmjF35.2720. The signals that drive the localisation of SNAREs are not well understood and can vary a lot: in some cases both the cytoplasmic tail and the transmembrane domain are of crucial importance, such as with syntaxin 5, and sometimes only the cytoplasmic tail bears the targeting signal, like the di-leucine motif used by endosomal syntaxins 7 and 8 [50].

When combining manual sequence alignments and analysis (looking for the presence of certain features, such as an absence of a transmembrane domain) and information on their localisation, we can only confidently annotate a few of the *Leishmania *SNARE proteins out of the 27 as orthologues of other known SNAREs. These are Qa SNARE LmjF32.0070, which shares 23% overall protein sequence identity with human syntaxin 5A and also localises to the Golgi and Qa SNARE LmjF28.1470 (along with related LmjF28.1480), which shares 38% and 39% identity in the SNARE domain with plasma membrane SNAREs such as *S. cerevisiae *Sso1p and human Syntaxin 1a, respectively and whose localisation in *Leishmania *shows it could be involved in a similar role. Finally, although we did not study its localisation, R syntaxin LmjF35.2120 can be identified thanks to its sequence features, as it shares 37% overall protein sequence identity with human Ytk6 and, like its orthologue, lacks a predicted transmembrane domain, but has a putative prenylation/palmitoylation site (Ytk6 is known to be able to mediate its own palmitoylation thanks to an activity located in the longin domain [51]). However, in general, there is poor sequence conservation, which presumably reflects the great sequence divergence of SNAREs between evolutionarily distant organisms. For instance, mammalian and yeast Bet1 are short (less than 150 amino acids) SNAREs from the Qc group and only share 17.5% amino acid sequence similarity between them. Two of the three short putative *L. major *Qc SNAREs (LmjF25.0090 and LmjF29.0630) share between 19 and 22%, respectively, amino acid identity with human Bet1. The remaining *L. major *short Qc SNARE (LmjF21.0560) did not return any significant BLAST result but shares 14.5% amino acid identity with Bet1-related mammalian GS15, also a Qc SNARE. Bet1 and GS15 are not the only proteins involved in vesicle fusion in the Golgi apparatus, but they are complexed to the same SNARE partners, although they act in an opposite directions for vesicle transport [52]. Hence, beyond the information given by the sequence, or even the location of the protein, functional studies are crucial to clearly assign a function and a name to each individual SNARE.

### Peculiarities of *L. major *SNAREs

The difficulty in clearly identifying SNARE homologues in *L. major *could also be due to the presence of a few novel SNAREs in *L. major*. This suggestion is supported by the fact that several members of the groups gave plant SNAREs as best BLAST hits (see Additional file 2), and plants such as *Arabidopsis thaliana *have a large number of SNAREs, some of which have a specialised and original role [17]. Our bioinformatic analysis of the predicted *L. major *SNAREs indeed reveals some peculiarities. First, *Leishmania *seem to be lacking several types of SNAREs including SNAP-25-like, large proteins bearing two SNARE motifs that can be found in fungi, plants and animals. Similarly, our analysis did not reveal the presence of any brevin-like proteins in the R group. However, *Leishmania *seem to possess a reasonably big SNARE repertoire (27) compared to the size of its genome and to the SNAREs numbers of yeast (21) or even metazoan like *Caenorhabditis elegans *(23) or *Drosophila melanogaster *(26) [11]. Also, the SNARE hypothesis implies an involvement of one member of each SNARE group into a complex and interestingly in this regard, with the exception of the R group, *Leishmania *possesses quite comparable numbers of proteins in the different groups. This suggests that the functions carried out by the missing SNAREs can be performed by structurally different ones in *Leishmania*. Indeed, *L. major *might also possess SNAREs not commonly found in other organisms. For instance, no homologue could be found for LmjF06.0820 (see Additional file 2), which is quite big for a SNARE (397 amino acids) but yields a predicted C-terminal transmembrane domain and, more importantly, a *bona fide *SNARE motif of the Qc type.

Two other putative *L. major *proteins bearing a SNARE domain were also special in the fact that they did not seem to contain any predicted transmembrane domain or lipidation site for attachment to the membrane (LmjF32.2160 and LmjF28.1480, see Additional file 2). Not only does LmjF32.2160 not possess a predicted membrane attachment motif, but it is also very larege (over 1300 amino acids) for a SNARE protein and, despite having an apparent R-SNARE motif, is unlikely to be functional. It has been shown that several syntaxin-binding proteins, such as tomosyn, possess a SNARE-like coiled-coil domain that would allow them to bind to and regulate a SNARE complexes [10]. Indeed, tomosyn regulates in space and time the release of neurotransmitters in neuronal tissues from mammals [53] and also *C. elegans *[54]. Interestingly, like LmjF32.2160, tomosyn is a large protein (over 1000 amino acids), lacking a C-terminal membrane anchor, and possessing a R-SNARE motif. However, LmjF32.2160 bears no significant sequence similarity and lacks the N-terminal WD40 repeats (generally involved in protein complexes formation) present in tomosyns. We could also find LmjF32.2160 homologues in two other kinetoplastids *Trypanosoma brucei *and *T. cruzi*, which suggests a conserved role for the protein in these parasites. However, it remains to be determined whether LmjF32.2160 is associated with the regulation of SNARE complexes. GFP-fusion of the predicted LmjF28.1480 showed that, indeed, it appeared to be soluble within the cytosol of cells (Fig. 7). However, as the *LmjF28.1480 *gene is located in the *Leishmania *genome in tandem with *LmjF28.1470*, which codes for a non-soluble Qa SNARE and only differs in its predicted version by the last 40 nucleotides of its 3' sequence, it might have arisen by gene duplication but have lost functionality. Indeed, a corresponding mRNA could be detected, but was longer than expected and contained several stop codons in the 5' UTR, suggesting that *LmjF28.1480 *could be a pseudogene. However, whether *Leishmania *can still possibly produce the short soluble version of LmjF28.1480 remains to be determined.

The *LmjF28.1470/LmjF28.1480 *genes represent a good illustration of both the conservation and additional complexity of the SNARE function in *Leishmania*. 5' mapping of the *LmjF28.1470 *mRNA-derived product amplified by PCR suggests that the protein can be produced, however a shorter version, potentially produced from an in-frame ATG, has a different cellular localisation. LmjF28.1470 is located near to the flagellar pocket, consistent with a role for a syntaxin 1 homologue, whereas N-terminally truncated LmjF28.1470 is found in the ER, where artificially produced syntaxin 1 chimeras have been found in mammalian cells. If only the full-length version of syntaxin 1 is produced, this raises the question as to what SNARE(s) would be involved in vesicular fusion in the ER in *Leishmania*, as no other member of the Qa group was found to localise to this compartment. Vesicular fusion in the ER could be mediated in a different way in *Leishmania *or, although in contradiction with the SNARE hypothesis, this function could be complemented by a member from different group. Even if the phylogenetic analysis of the SNARE domains was quite clear, there could also have been a misannotation of one of the Qa members, or it might be so divergent that it was not found in the genome database. The syntaxin 1 repertoire seems to be increased by alternative splicing in other organisms [55,56] with tissue-specific expression profiles and functions. Thus, another hypothesis is that *Leishmania *could produce both LmjF28.1470 isoforms to act in different compartments as a part of different SNARE complexes. In any case, our findings only lay the groundwork for future functional studies and the analysis of the native SNARE proteins of *Leishmania *could possibly unveil a few surprises.

## Conclusion

We have identified 27 SNARE domain-containing putative proteins encoded in the genome of *L. major*, which could be classified into the four major groups of SNAREs based on the sequence of their SNARE motif. The SNARE repertoire is more than can be found in organisms such as *S. cerevisiae *and *C. elegans*. Additionally, we identified several putative regulatory proteins of the SNARE complexes. Systematic GFP-fusion and *in vivo *expression of the members of the Qa/syntaxin group also revealed targeting of some to specific intracellular compartments. Together, these data suggest that *L. major*, and kinetoplastids in general, have a well-developed vesicle-fusion machinery of which features appear to be conserved throughout the various eukaryotic lineages. The amenability of these proteins to N-terminal GFP fusion should make some of the proteins useful markers for specific intracellular compartments that are currently lacking for the cell biology studies of kinetoplastid parasites. The SNAREs are generally very divergent in sequence and, for most it has not been possible to ascribe a function in *Leishmania *based solely on sequence similarity. To this end there is a need for targeted functional studies, especially because *L. major *also has some predicted SNAREs that appear very peculiar in structure and so analyses of these could give insights into new functions and cellular mechanisms. Our data suggest that the expression of certain SNAREs may be stage-regulated in *Leishmania*, and as the trafficking machinery is important for the virulence of these parasites, future studies may reveal some interesting opportunities to discover ways of interfering with critical pathways.

## Methods

### Genome searches

We retrieved all previously identified SNARE proteins sequences from *Saccharomyces cerevisiae *and *Homo sapiens *genomes from the NCBI protein database [57]. These sequences were used in BLASTP searches against the *Leishmania major *genome at GeneDB [39]. BLAST hits having log E values of < 0.1 were used for further analysis. The Pfam motif search utility within GeneDB was also used to detect annotated SNARE domain-containing proteins. *S. cerevisiae *and *H. sapiens *sequences from each SNARE group (Qa, Qb, Qc and R) were used to create an Hidden Markov Model (HMM) profile for each of the subgroups, using the HMMER package [58], and each profile was used to search against the GeneDB *L. major *predicted proteins database (10/05/05 release) to identify any additional candidate. As a last control, the SNARE domain only of each identified *L. major *sequence was used for a BLASTP search of the *L. major *GeneDB database to try to identify any other possible candidate.

All the obtained *L. major *protein sequences were searched against the Pfam [59] and PROSITE [60] domain databases to confirm their homologies and locate the identified domains. Domains used for identification were Pfam domains PF05739 (SNARE), PF05008 (V-SNARE), PF00957 (Synaptobrevin) and PROSITE domains PS50192 (SNARE), PS50892 (V-SNARE), PS50859 (longin).

Putative functions were assigned to the identified predicted proteins by BLASTP search against the NCBI nr database when we obtained a good score (E value usually less than 10^-20^) with a corresponding functionally characterised protein from another eukaryote and a reciprocal BLASTP search in the GeneDB database with the characterised protein sequence also gave the *L. major *protein as the best match.

### Sequences alignments and phylogenetic analyses

Amino acid sequences of the SNARE domain only were aligned using the Clustal W algorithm with AlignX program included in the Vector NTI 7.1 package (Invitrogen). The set of data was exported as a multiple sequence file (MSF) format and used to build a phylogenetic tree with the MEGA3 software [61,62]. The tree was made using the UPGMA (Unweighted Pair Group Method using Arithmetic averages) algorithm and Poisson-corrected amino acid distance was used. The tree was then unrooted and radiation representation was used to visualise it. The reliability of clustering patterns in the tree was tested by bootstrapping (1000 pseudoreplicates).

### Sequences analyses

Transmembrane domains and protein topologies were predicted using the Sosui algorithm [63,64]. Secondary structures in the N-terminal parts of syntaxins were predicted using the Jpred algorithm [65,66]. Putative prenylation sites were predicted using the PrePS prenylation prediction suite [67,68] and putative palmitoylation sites were identified using the CSS-Palm algorithm [69], with a cut-off value of 4 [70].

### Parasites

*L. major *(MHOM/JL/80/Friedlin) promastigotes were grown in modified Eagle's medium (designated complete HOMEM medium) with 10% (v/v) heat-inactivated foetal calf serum at 25°C as described previously [71]. Amastigotes were isolated from mouse lesions as described previously [72].

### Cloning and expression of putative *Leishmania *syntaxin genes

The genes coding for putative syntaxins were amplified from *L. major *DNA using primers in which restriction sites used for subsequent cloning were incorporated (Table 2, in bold). The obtained fragments (sizes displayed in Table 2) were cloned into the pNUS-GFPnNeo vector [41], allowing the in-frame insertion of the gene of interest at the 3' end of the *GFP *gene. Different cloning strategies and restriction sites were used according to the ones already present in the genes, *Bgl*II/*Kpn*I for *LmjF29.0070*, *Bgl*II/*Eco*RV for *LmjF28.1470*, *LmjF28.1480*, *LmjF19.0120 *and *LmjF32.0070*, *Bgl*II/*Xho*I for *LmjF33.1340 *and *Kpn*I/*Eco*RV for *LmjF35.2720*. The digested fragments were ligated into the pNUS-GFPnNeo vector previously digested by the appropriate enzymes. Log phase *L. major *promastigotes were transfected with 20 μg of plasmid and selected with 50 μg/ml of G418. Drug resistant populations were checked by PCR for the presence of the plasmids.

### RNA extraction and mapping of 5'-end using RT-PCR

Total RNA was extracted from ~10^8 ^promastigotes or amastigotes by the phenol-chloroform-guanidine isothiocyanate method using the TRIzol reagent (Invitrogen) according to the protocol supplied by the manufacturer. Samples were then subsequently treated with RQ1 RNase-free DNase (Promega) to remove any contaminating DNA. After a phenol-chloroform extraction, RNA was precipitated and 5 μg used to synthesise first strand of cDNA with SuperScript III reverse transcriptase (Invitrogen) following the manufacturer's instructions. One-tenth of the final material was used as a template for separate PCR reactions using an upper strand splice-leader primer (OL1760, Table 3) and specific 3' reverse primers for the seven *L. major *Qa genes (see Additional file 2). 1% of each reaction was used as a template for a round of semi nested-PCR amplification using internal primers: splice-leader oligonucleotide (OL1760) was still used as the 5' primer, together with specific internal 3' reverse primers for the genes (Table 3). Products of both series of reactions were resolved on a 2% agarose gel and fragments from selected genes were, once excised from the gel, cloned into the pGEM-T vector (Promega), allowing them to be sequenced to precisely map the 5' trans-splicing acceptor sites.

### Fluorescent staining of cells

10^7 ^*L. major *promastigotes were harvested by centrifugation and washed once in serum-free HOMEM. For FM4-64 labelling, the cells were incubated with 40 μM of FM4-64 (from a 12 mM stock solution in DMSO; Invitrogen) for 15 min at 4°C and then washed in fresh medium and incubated for various times at 25°C. Cells were then washed in cold phosphate-buffered saline (PBS) and processed for microscopy.

For immunofluorescence analysis, cells were processed as described previously [73]. Briefly, after fixation, the solution was adjusted to 0.1% Triton X-100 and incubated for 10 min; glycine (0.1 M) was added for 10 min. After centrifugation, the cells were resuspended in PBS and allowed to dry onto glass slides. Primary antibodies were incubated at the appropriate dilution (1/200 for mouse anti-*Lm*LCB2 [49], 1/2000 for rabbit anti-*Tb*Rab1 [44], 1/1000 for rabbit anti-*Tb*V-H^+^-PP [45], 1/500 for rabbit anti-*Ld*PFK [46]) in PBS with 0.1% BSA for 30 min, followed by three washes in PBS and subsequent incubation with fluorochrome-conjugated secondary antibodies. Cells were viewed with a Zeiss UV microscope and images were captured by an Orca-ER camera (Hammamatsu) and Openlab software v 4.0.3 (Improvision). The "volume deconvolution" module from Openlab was used to remove background staining when using the anti-*Tb*Rab1 antibody.

## Authors' contributions

SB conceived the study in consultation with JCM, carried out the experiments, analysed the data and drafted the paper. GHC and JCM helped to analyse the results and to write the manuscript.

## Supplementary Material

Additional file 1 figure S1 – Alignments of the SNARE domain of putative SNAREs from *Leishmania *with characterised mammalian or yeast members of each group: Qa (A), Qb (B), Qc (C) and R (D). figure S2 – Schematic representation of putative SNARE domain-containing *L. major *proteins. Figure S3 – Western blot analysis of the cell lines expressing GFP-fused members of the Qa group. Click here for file

Additional file 2 table S1 – Predicted SNARE domain-bearing proteins from *L. major *and their properties Click here for file

## Figures and Tables

**Table 1:** Putative *L. major *orthologues of mammalian and yeast SNARE-interacting proteins.

	**SNARE-interacting protein**	***L. major*** **orthologue**	**E value**	**AA**
SNAP/NSF	α/β-SNAP	LmjF20.1690	3.00E-29	282
	α/β-SNAP	LmjF32.2890	1.30E-26	291
	γ-SNAP	LmjF34.0540	3.10E-07	389
	NSF	LmjF20.0810	1.20E-135	738
				
SM proteins	SLY1	LmjF26.2360	4.80E-61	682
	VPS45	LmjF36.2230	1.30E-65	559
	VPS45	LmjF36.0460	2.80E-56	617
	VPS33	LmjF25.2180	1.40E-21	597
	Sec1/Munc18	LmjF26.2260	3.70E-39	742
				
Others	GATE-16	LmjF19.1630	2.20E-26	125
	EpsinR	LmjF25.0670	9.00E-20	594
	Vsm1p	LmjF01.0610	1.00E-35	243
	Vtc1	LmjF14.1040	1.20E-18	180
	Vtc2, 3 or 4	LmjF09.0220	3.90E-26	813

List of identified putative *L. major *SNARE-interacting orthologues with their BLAST E value and their predicted size in amino acids (AA).

**Table 2:** Primers used to amplify *L. major *Qa SNAREs.

**Primer name**	**Primer sequence**	**Gene amplified**	**Fragment size (bp)**
OL1970	CGT**AGATCT**ATGCAGGAAATGGGGG	5' of *LmjF29.0070*	
OL1971	CTT**GGTACC**TCAGTTCATGAGCAGA	3' of *LmjF29.0070*	755
OL2150	AAG**AGATCT**ATGGATCGTCTTCCAC	5' of *LmjF28.1470*	
OL2038	AAG**AGATCT**ATGGCCGAGCTGCAGC	5' of short *LmjF28.1470/LmjF28.1480*	
OL2039	GTC**GATATC**CTACAGAACCGTAAAC	3' of *LmjF28.1470*	864/1107
OL2048	ACG**GATATC**TCAGTCCTCCCAGGCC	3' of *LmjF28.1480*	801
OL2040	AAG**AGATCT**ATGCCCTTAGAGAATG	5' of *LmjF19.0120*	
OL2041	ACG**GATATC**TCATCGGGTGAGAACC	3' of *LmjF19.0120*	690
OL2042	AAG**AGATCT**ATGCGTTTGACGCAGC	5' of *LmjF32.0070*	
OL2043	ACGG**ATATCT**TAGCGCACCACGAGG	3' of *LmjF32.0070*	738
OL2044	AAG**AGATCT**ATGGAACTGAAAGTCA	5' of *LmjF33.1340*	
OL2045	ACG**CTCGAG**TCACATAAGAATGCAG	3' of *LmjF33.1340*	828
OL2046	ACG**GGTACC**ATGGCGACTCGCGACC	5' of *LmjF35.2720*	
OL2047	ACG**GATATC**TCAGGTAATGGCCTTG	3' of *LmjF35.2720*	909

Specific primers used to amplify the specified *L. major *genes to give products of which the sizes are indicated on the right. Restriction sites used for cloning are in bold.

**Table 3:** Primers used for semi-nested RT-PCRs.

**Primer name**	**Primer sequence**	**Target**
OL1760	AACTAACGCTATATAAGTATCAGTTTCTGTACTTTATTG	*L. major *splice-leader sequence
OL2147	CTCCTTCCGGACAACAGTGCCGGTGC	3' of *LmjF28.1480*
OL2164	CGGCGGTGCTTTTGCA	3' of *LmjF28.1470*
OL2165	CTACAATCATGACGAT	3' of *LmjF19.0120*
OL2166	GATGGCAAGCATGATC	3' of *LmjF29.0070*
OL2167	GAATAGCATTGCAAAC	3' of *LmjF32.0070*
OL2168	GCTCTGATTCTCGCGA	3' of *LmjF33.1340*
OL2169	CGAGGGCTATCATCAA	3' of *LmjF35.2720*

Primers used to amplify syntaxin-specific products from *L. major *promastigotes cDNA.
