# Supplementary material for: The SNARE protein family of Leishmania major
Source: BMC Genomics. 2006 Oct 6;7:250. doi: 10.1186/1471-2164-7-250 (PMC1626469; doi:10.1186/1471-2164-7-250)
Supplement: Additional file 1 — figure S1 – Alignments of the SNARE domain of putative SNAREs from Leishmania with characterised mammalian or yeast members of each group: Qa (A), Qb (B), Qc (C) and R (D). figure S2 – Schematic representation of putative SNARE domain-containing L. major proteins. Figure S3 – Western blot analysis of the cell lines expressing GFP-fused members of the Qa group. [file 1471-2164-7-250-S1.doc]

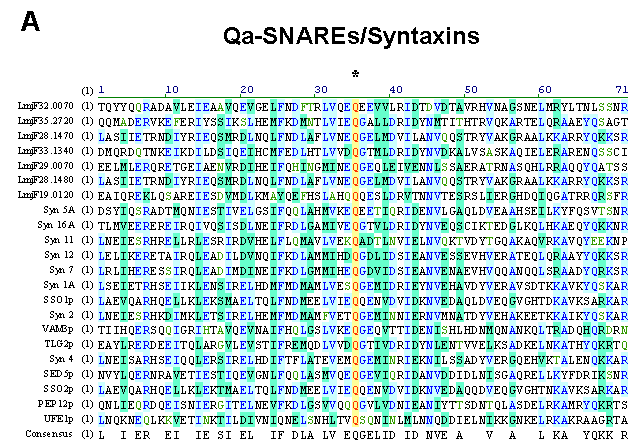


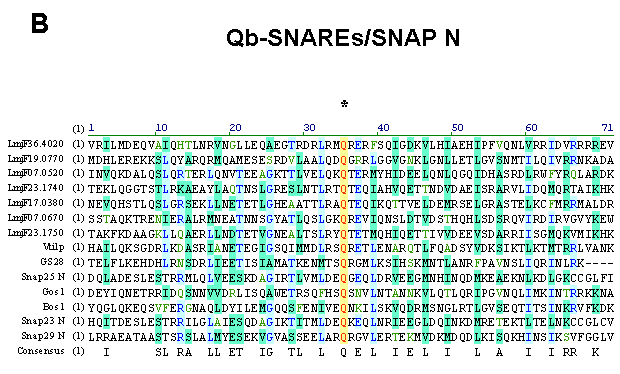


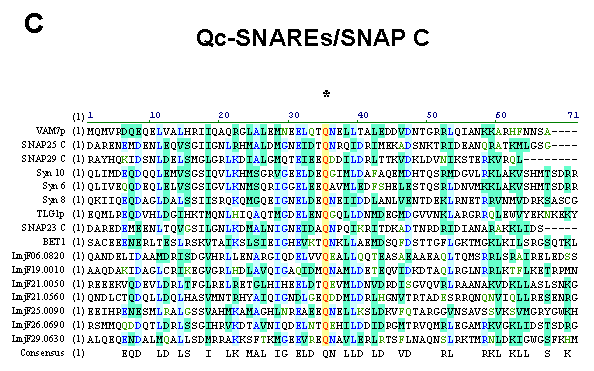


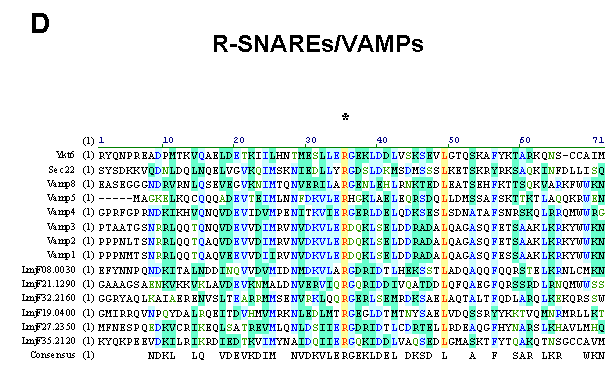


Figure S1. Alignment of the SNARE domains from *Leishmania* along with selected yeast or mammalian members of each SNARE groups (A, Qa-SNAREs; B, Qb-SNAREs; C, Qc-SNAREs; D, R-SNAREs). Identical amino acids are shown in red letters on a yellow background, conservative in dark blue letters on a light blue backgound, blocks of similar amino acids are shown on a green background and weakly similar are shown in green letters. The asterisk indicates the central Q or R residue. Origin of sequences used and their Genbank accession numbers are as follows: Qa group - Syn 5A (human, NP_003155), Syn 16A (human, NP_001001433), Syn 11 (human, NP_003755), Syn 12 (human, NP_803173), Syn 7 (human, NP_003560), Syn 1A (human, NP_004594), Syn 2 (human, NP_919337), Syn 4 (human, AAG40313), Sso1p (yeast, YPL232W), Sso2p (yeast, YMR183C), Vam3p (yeast, YOR106W), Tlg2p (yeast, YOL018C), Sed5p (yeast, YLR026C), Pep12p (yeast, YOR036W), Pep12p (yeast, YOR075W); Qb group - SNAP23 (human, CAG33493), SNAP25 (human, CAB42860), SNAP29 (human, CAG30468), Gos28 (human, NP_001007026), Gos1p (yeast, NP_011832), Bos1p (yeast, CAA97636), Vti1p (yeast, Q04338); Qc group - SNAP23, SNAP25, SNAP29 (human, see above), Syn 6 (human, NP_005810), Syn 8 (human, NP_004844), Syn 10 (human, AAC05087), BET1 (human, EAL24137), Vam7p (yeast, YGL212W), Tlg1p (yeast, YDR468C); R group - Vamp1 (human, CAA88760), Vamp2 (human, AAF15551), Vamp3 (human, NP_004772), Vamp4 (human, AAC24032), Vamp5 (mouse, AAC95282), Vamp8 (human, NP_003752), Ykt6 (human, CAG46805), Sec22 (yeast, P22214).

Figure S2. Structure of predicted SNARES from *L. major*


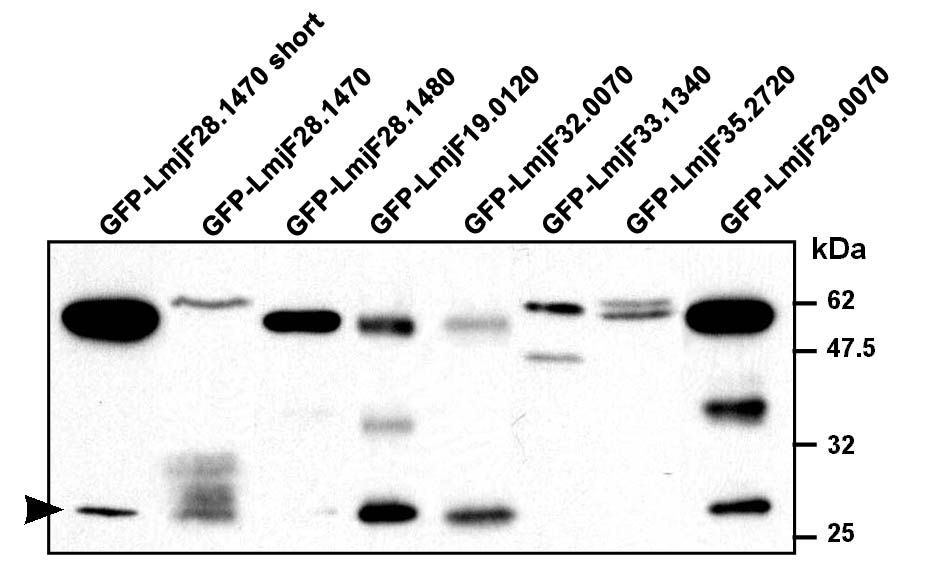


Figure S3. Western blot analysis of the *L. major* cell lines expressing GFP-fused members of the Qa group. Cell extracts from 2.10^7^ promastigotes from each cell line were analysed with an anti-GFP monoclonal antibody (Santa Cruz), followed by an anti-mouse horseradish peroxidase-conjugated secondary antibody (Promega) and proteins were revealed with an ECL substrate kit (Pierce). The anti-GFP antibody gave no significant signal when used in similar conditions on wild-type cell extracts (data not shown).

The expected sizes of the fusion proteins (in kDa) are as follows: GFP-LmjF28.1470 short, 58.9; GFP-LmjF28.1470, 67.3; GFP-LmjF28.1480, 57.5; GFP-LmjF19.0120, 53.2; GFP-LmjF32.0070, 55; GFP-LmjF33.1340, 58.9; GFP-LmjF35.2720, 62.7; GFP-LmjF29.0070, 56.7.

All cell lines were found to express GFP-fusion proteins consistent with the predicted size, with some occasional additional bands probably denoting degradation products, including a band corresponding to the size of the GFP only (27 kDa, arrowhead). However, the presence of such a degradation product is not detrimental for the interpretation of the localisation of the GFP fusions, as the fluorescence patterns observed for the cell lines displaying such a band were generally not the same (i.e. GFP-LmjF19.0120, endo-lysosomal system; GFP-LmjF19.0120, Golgi; GFP-LmjF28.1470 short, endoplasmic reticulum), thus there is no correlation between the presence of the 27 kDa band and a specific fluorescence signal. Nevertheless, it is interesting to note that the two cell lines that seem to be more prone to degradation of their GFP-fusion protein (GFP-LmjF19.0120 and GFP-LmjF32.0070) are expressing syntaxins that have not been detected by RT-PCR in the promastigote stage (Fig. 2), suggesting that they could be specifically degraded by the cells as a regulation, as they are normally not (or weakly) expressed in this stage of the parasite.
