# Supplementary material for: The SNARE protein family of Leishmania major
Source: BMC Genomics. 2006 Oct 6;7:250. doi: 10.1186/1471-2164-7-250 (PMC1626469; doi:10.1186/1471-2164-7-250)
Supplement: Additional file 2 — table S1 – Predicted SNARE domain-bearing proteins from L. major and their properties [file 1471-2164-7-250-S2.doc]

| **SNARE group** | ***L. major* protein** | **E value** | **BLAST hit** | **Size (AA)** | **transmbne domain** | | **SNARE motif** | **Nter features** | | **Rev BLAST** | |
| --- | --- | --- | --- | --- | --- | --- | --- | --- | --- | --- | --- |
|  | LmjF19.0120 | 8.E-10 | syntaxin 12 [*Mus musculus*] | 229 | 205-226 |  | 138-200 | Habc |  | LmjF35.2720 | |
|  | LmjF28.1470 | 1.E-18 | syntaxin 1B [*Limulus polyphemus*] | 368 | 306-328 |  | 236-288 | Habc |  | LmjF28.1470/80 | |
|  | LmjF28.1480 | 1.E-16 | syntaxin 1B [*Limulus polyphemus*] | 266 | no |  | 155-217 | Habc |  | LmjF28.1470/80 | |
| **Qa** | LmjF29.0070 | 2.E-11 | Vam3p [*Aspergillus oryzae*] | 254 | 234-254 |  | 166-228 | Habc |  | LmjF29.0070 | |
|  | LmjF32.0070 | 1.E-20 | syntaxin 5A [*Mus musculus*] | 307 | 283-305 |  | 219-281 | Habc |  | LmjF32.0070 | |
|  | LmjF33.1340 | 4.E-15 | syntaxin 16 [*Mus musculus*] | 275 | no | prenyl/palm | 208-270 | Habc |  | LmjF35.2720 | |
|  | LmjF35.2720 | 4.E-21 | syntaxin 16 [*Gallus gallus*] | 302 | 277-299 |  | 212-274 | Habc |  | LmjF35.2720 | |
|  | LmjF07.0520 | 2.E-06 | SNARE 13 [*Oryza sativa*] | 236 | 206-228 |  | 138-203 |  |  | LmjF07.0520 | |
|  | LmjF07.0670 | 3.E-02 | Vti1b [*Mus musculus*] | 166 | 142-164 |  | 75-133 | Habc |  | LmjF23.1750 | |
|  | LmjF17.0380 | 2.E-04 | SNARE 11 [*Oryza sativa*] | 275 | 248-270 |  | 180-245 |  |  | LmjF17.0380 | |
| **Qb** | LmjF19.0770 | 8.E-08 | Membrin 11 [*Arabidopsis thaliana*] | 235 | 214-235 |  | 155-211 |  |  | LmjF19.0770 | |
|  | LmjF23.1740 | 2.E-04 | v-SNARE 11 [*Arabidopsis thaliana*] AtVTI11 | 250 | 229-250 |  | 161-228 | Habc |  | LmjF23.1740/50 | |
|  | LmjF23.1750 | 3.E-06 | Vti1a [*Mus musculus*] | 338 | 281-302 |  | 218-280 | Habc |  | LmjF23.1750 | |
|  | LmjF36.4020 | 2.E-04 | Gos12 [*Arabidopsis thaliana*] | 276 | 257-276 |  | 198-257 |  |  | LmjF36.4020 | |
|  | LmjF06.0820 |  | none | 397 | 372-394 |  | 309-371 |  |  |  |  |
|  | LmjF19.0010 | 1.E-05 | syntaxin 8 [*Gallus gallus*] | 295 | 271-293 |  | 205-267 | Habc |  | LmjF26.0690 | |
|  | LmjF21.0050 | 3.E-05 | SYP61 [*Arabidopsis thaliana*]/synt6 | 233 | 213-231 |  | 147-209 | Habc |  | LmjF26.0690 | |
| **Qc** | LmjF21.0560 | 1.E-15 | none | 96 | 75-96 |  | 13-75 |  |  |  |  |
|  | LmjF25.0090 | 3.E-01 | predicted Bet1 [*Canis familiaris*] | 120 | 96-117 |  | 32-92 |  |  | LmjF29.0630 | |
|  | LmjF26.0690 | 2.E-11 | SYP61 [*Arabidopsis thaliana*]/synt6 | 250 | 231-250 |  | 164-226 |  |  | LmjF26.0690 | |
|  | LmjF29.0630 | 3.E-04 | Bet1 homolog [*Mus musculus*] | 139 | 116-137 |  | 23-111 |  |  | LmjF29.0630 | |
|  | LmjF08.0030 | 9.E-13 | VAMP7C [*Arabidopsis thaliana*] | 215 | 185-207 |  | 123-185 | longin domain | | LmjF27.2350 | |
|  | LmjF19.0400 | 1.E-10 | Sec22 [*Saccharomyces cerevisiae*] | 280 | 259-280 |  | 192-254 |  |  | LmjF19.0400 | |
| **R** | LmjF21.1290 | 9.E-11 | VAMP4 [*Homo sapiens*] | 257 | 219-239 |  | 154-216 | longin domain | | LmjF08.0030 | |
|  | LmjF27.2350 | 3.E-18 | VAMP1 [*Homo sapiens*] | 220 | 192-214 |  | 129-191 | longin domain | | LmjF08.0030 | |
|  | LmjF32.2160 | 1.E-01 | none | 1305 | no |  | 1240-1299 |  |  |  |  |
|  | LmjF35.2120 | 3.E-34 | YKT6 [*Homo sapiens*] | 205 | no | prenyl | 141-198 | longin domain | | LmjF35.2120 | |

Table S1. Predicted SNARE domain-bearing proteins from *L. major* and their properties

Identified *L. major* SNARE domain-containing proteins are listed together with their best corresponding BLAST hit along with the E values. When reverse BLAST gave the original *L. major* protein, its name was underlined (Rev BLAST column). The putative properties of these predicted proteins are displayed: their size in amino acids (AA), the presence and location of a transmembrane domain (Transmbne domain) or a putative prenylation (prenyl)/palmitoylation (palm) site, the presence and location of a SNARE domain and N terminal features such as a triple helical region (Habc) or a longin domain
